# Ethnopharmacobotany and Diversity of Mediterranean Endemic Plants in Marmilla Subregion, Sardinia, Italy

**DOI:** 10.3390/plants11223165

**Published:** 2022-11-18

**Authors:** Emma Cocco, Delia Maccioni, Enrico Sanjust, Danilo Falconieri, Emmanuele Farris, Andrea Maxia

**Affiliations:** 1 Laboratory of Economic and Pharmaceutical Botany, Department of Life and Environmental Sciences, Università degli Studi di Cagliari, V.le S. Ignazio da Laconi 13, 09123 Cagliari, Italy; 2 Department of Biomedical Sciences, University of Cagliari, Cittadella Universitaria, SS 554 bivio Sestu, 09042 Monserrato, Italy; 3 Istituto Tecnico Industriale Statale “Michele Giua”, Via Montecassino, 09100 Cagliari, Italy; 4 Department of Chemical, Physical, Mathematical and Natural Sciences, Università degli Studi di Sassari, Via Piandanna 4, 07100 Sassari, Italy

**Keywords:** ethnobotany, Marmilla, Mediterranean endemic medicinal plants, plant diversity and conservation, Sardinia, traditional botanical knowledge

## Abstract

Human populations in various regions across the world exploit the medicinal properties of plants to treat a wide variety of diseases. Areas with both high rates of endemic taxa and persisting traditional uses of the local botanical resources are key sites for the investigation of Traditional Botanical Knowledge (TBK). Commonly, in these areas, information regarding the medicinal properties of native plants has been transmitted orally from generation to generation, however, a rapid decline in this knowledge has been observed, which can be attributed to socio-economic changes in recent years. The Mediterranean basin is one such site, where human history is intimately entwined with nature. The unique geographical situation and unrivaled environmental heterogeneity of the area, have allowed both the development of diverse civilizations as well as providing the basis for the evolution of extraordinary biodiversity. The Mediterranean basin can therefore be considered a global hotspot of endemic vascular plants, and of traditional knowledge of medicinal and aromatic species. This study researches the historical subregion of Marmilla (central-southern Sardinia, Italy), which was chosen because of its specific cultural and demographic characteristics: i.e., prolonged isolation and extreme longevity of the inhabitants of the area. Semi-structured interviews were conducted with 145 people from the region, and 137 medicinal plants belonging to 62 families were identified, of which around 57,3% were taxa exclusive to the Mediterranean Basin. Findings showed that the most used parts of the plant were the leaves (49%), while as far as preparations are concerned, decoction (50%) was the most used to prepare medicinal formulations, making this the highest number of medico-botanical taxa reported in a study carried out in Sardinia using a similar methodology. In addition, this study contributes towards preventing the loss of TBK by documenting the medicinal traditions, passed down orally for centuries, in the words of the participants, shedding new light on the traditional knowledge of the inhabitants of the island. The findings lay the foundations for future applied studies in the fields of phytotherapy and phytochemical investigation.

## 1. Introduction

The variety of environments on Earth and the complex relationships among plants themselves and with the entire ecosystem led to the extreme biological diversity of these organisms [1,2]. As a result, highly diversified adaptation strategies have evolved, reflected either in phenotypic or chemical variability [3,4]. This is particularly true for medicinal plants, whose active ingredients have been used for centuries in folk tradition. Even today, they represent an essential source of study in the phytochemistry, biochemistry, and pharmacology fields, eventually providing new molecules to be tested in therapeutics [5,6]. However, in recent decades the modernization of society has limited the use of orally transmitted folk medicine, causing an intergenerational erosion of Traditional Botanical Knowledge [7,8]. Therefore, those areas where customs and traditions persist are crucial to preserving TBK’s long history [9]. 

In this context, Sardinia, the second-largest island in the Mediterranean basin, plays a relevant role. The Mediterranean Basin hosts ca. 25,000 vascular plant species, of which 13,000 (4.3% of the global vascular flora) are considered exclusive [10]. Only the hotspots in the Tropical Andes (6.7%) and Sundaland (5.0%) have higher percentages of exclusive taxa [10]. The Mediterranean basin, recently defined as a complex, multi-hierarchical system of islands-within-islands [11] because of its pronounced geographical and ecological patchiness, has an original and ongoing history of the evolution of plant populations, communities, and landscapes [12]. In this context, the combinations of several evolutionary factors, such as richness in microhabitats and geographical isolation over the long period, have promoted further diversification that makes Sardinia a hotspot within a hotspot [13,14], with a high rate of endemism (341 endemic taxa over ca. 2300 native vascular plants). In addition, endemic vascular plants thrive mainly in harsh environments where they often produce a wide variety of secondary metabolites [12] which give them specialized properties. For this reason, the Mediterranean basin is also a hotspot of medicinal and aromatic plants, where the study of the plant/human relationship acquires greater value [15].

Interestingly, the same evolutionary factors influencing plant diversity have also affected the Sardinian human population. The geographical isolation, especially in inland areas with a limited population, has allowed the association of an extreme richness of biodiversity and endemism with a strong TBK and a consequent high potential for the medicinal use of native plants [16]. Although the prolonged isolation limited the loss of local knowledge, the inner areas currently face migration and depopulation phenomena, which lead to progressive TBK erosion [17,18]. At the same time, plant species extinction, which is already on the rise, is expected to increase dramatically in light of ongoing climate change, threatening biodiversity, and the ecosystem [13,19]. 

These factors have made Sardinia an ideal place for the study of ethnobotanical traditions, which is why several studies have been published about the island [20,21,22]. However, only a few of these reported the knowledge of entire subregions [23,24,25], characterized by common historical and cultural paths, while existing research most often focuses on a single village, providing fragmentary information [26,27]. In particular, the Marmilla subregion has not yet been thoroughly examined, despite being one of the depopulation areas on the island, which endangers TBK transmission. In fact, its weakness in the economic structure, and its geographic isolation results in high emigration rates and an older demographic profile. 

Furthermore, the Marmilla subregion (central-southern Sardinia), provides a good setting for studying TBK dynamics since it either lies in or borders the Extended Blue Zone (EBZ) of the island of Sardinia [28]. The EBZ and RBZ (Restricted Blue Zone) are extreme longevity areas that hold the secret to Sardinia’s healthy population, collectively called the Sardinian Blue Zone (SBZ) [29]. Although the factors underlying this exceptional longevity remain unknown, genetic and environmental factors are believed to drive these longevity hotspots. The SBZ is an excellent example, in particular, of how geographical isolation resulted in genetic isolation as well as the preservation of homogeneous lifestyle, dietary, physical activity, and traditional knowledge.

The geographical isolation present in Sardinia produced well-defined subregions in terms of customs and traditions, which are also reflected in language differences. Therefore, the present study focuses on the Marmilla subregion because, despite its unique characteristic, it has never been thoroughly examined. The main objectives are i) to record the medicinal plants used, by monitoring the plant biodiversity of the territory; and ii) to codify the ethnobotanical use, preparation, and effects of the medicinal plants, preventing the loss of cultural heritage. Therefore, this work provides a valuable contribution to the ethnobotanical uses of Mediterranean endemic plants. 

## 2. Results

**Figure 1:**
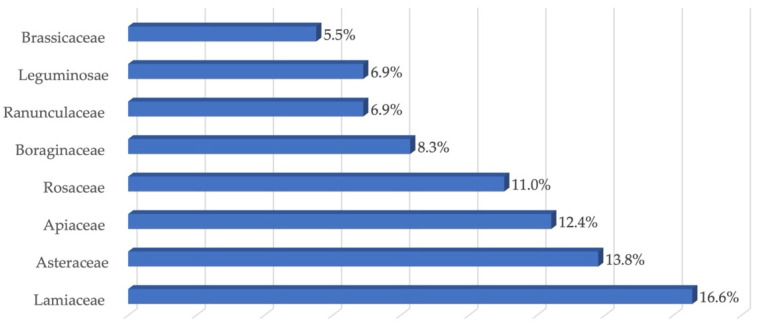
Percentage of abundance of taxa per family.

The results of the interviews were processed and presented graphically in Table 1. The species were firstly divided by family, then, for each species, several data were provided: vernacular name, part of the plant used and preparation, and finally the reported effect of the herbal drug. Emergent from the research was the use, in the Marmilla area, of 137 medicinal plants, belonging to 62 families. The family with the most significant number of taxa was Lamiaceae, followed by Asteraceae, Apiaceae, Rosaceae, and Ranunculaceae (Figure 1).

It is interesting to note that the majority (54.4%) of the local medicinal flora is Mediterranean endemic taxa (endemics *sensu lato*, [30] whereas four of the collected plants are endemic *sensu stricto* (*Dipsacus ferox* Loisel., *Helichrysum microphyllum* (Willd.) Cambess. Subsp. *Tyrrhenicum* (Bacch., Brullo & Giusso) Herrando, J.M. Blanco, L. Sáez Galbany, *Scrophularia trifoliata* L. and *Stachys glutinosa* L.), corresponding to 2.9% of the local medicinal flora. So overall, even if endemics *sensu stricto* represents 15% of the whole native flora of the island [31], it is striking that 57.3% of the local medicinal flora of Marmilla is represented by Mediterranean endemic taxa.

**Table 1:** List of the emerged species: scientific and local name, part of the plant used, preparation, and Folk therapeutic uses.

n°	Family	Taxa	Local Name	Part Used	Preparation	Folk Therapeutic Uses
1	Adoxaceae	* Sambucus nigra * L.	Sammucu	Le; Fl	De	Emollient and decongestant in case of edema
2	Amaranthaceae	* Chenopodium vulvaria * L.	Cadoni budésu	AP	Inf	Emmenagogue, anthysteric, antirheumatic
3	Amaryllidaceae	* Narcissus tazetta * L.	Narcisu	Le	De	Antispasmodic, sedative, emetocathartic, emmenagogue
4	Anacardiaceae	* Pistacia lentiscus * L.	Ollestincu	Re	IU	Painkiller, expectorant [32], intestinal astringent, stomachic, hemostatic
5	Apiaceae	* Bupleurum fruticosum * L.	Linna budescia	Le	De	Astringent, vulnerary
6		* Daucus carota * L.	Pistinaga	Se	De	Carminative and revitaminizing
7		* Eryngium campestre * L.	Spin’e corra	Rt	De	Diuretic, cholagogue, emmenagogue
8		* Ferula communis * L.	Fèurra	Fl	Co	Antirheumatic
9		* Foeniculum vulgare * Mill.	Fenoghu	Fr; Rt	Inf	Adjuvant for slimming diets [33], expectorant
				Fr	IU	Against halitosis and anorexia
10		* Helosciadium crassipes * W.D.J.Koch ex Rchb.	Appiu	Le	De	Against bronchial and pharyngeal catarrhs
					IU	Antiscorbutic
11		* Oenanthe fistulosa * L.	Appiu burdu	Le	Fu	Sedative, expectorant
12		* Scandix pecten-veneris * L.	Erba de agullas	Rt	De	Anti-inflammatory, astringent, eupeptic gastric dyspepsia, cystitis, nephritis, pyelitis
13		* Thapsia garganica * L.	Feruledda	Lt	EU	Vesicatory, against lumbago, ischialgia, and rheumatic or neuralgic affections
14	Apocynaceae	* Nerium oleander * L.	Launaxi	Le	De	Against skin lesion
15	Araliaceae	* Hedera helix * L.	Édera	Le	De	Against neuritis and neuralgia of gouty or rheumatic origin
16	Asparagaceae	* Muscari comosum * (L.) Mill.	Cibudda de colorus	Bu	De	Emollient
17		* Prospero autumnale * (L.) Speta	Lillixeddu	Cas	De	Diuretic, thin broncho-pulmonary secretion, increased arterial tension
18		* Ruscus aculeatus * L.	Spinadoppis	AP	Inf	Diuretic
19	Aspleniaceae	* Asplenium onopteris * L.	Filixi	AP	De	Expectorant, emollient, adjuvant bronchial affections
20		* Asplenium trichomanes * L.	Sfarzi de rana	AP	De	Expectorant emollient, adjuvant bronchial affections
21	Asphodelaceae	* Asphodelus ramosus * L.	Kadrilloni	UP	Ca	Chilblain’s treatment, adsorbent in case of intoxication [24]
				Fl	DI	
22	Asteraceae	* Achillea millefolium * L.	Erba de feridas	Fl	Inf	Cholagogue, crubs uterine bleeding relieves hemorrhoids and pulmonary disorders
23		* Artemisia arborescens * (Vaill.) L.	Sentsu	Le; Fl	Co	Against pleurisy, bronchitis, headaches
				Le	De	Diuretic
24		* Bellis perennis * L.	Sittsia	WP	De	Against uterine hemorrhages
					Inf	Treatment of pleurisy and upper respiratory tract disease
					Co	Against bruises, sprains, and boils
25		* Calendula arvensis* (Vaill.) L.	Erba de froris	Le	De	Antiphlogistic, astringent, cleansing, diuretic, emmenagogue, emetocathartic, sedative, topic, and sudorific
26		* Carlina gummifera * (L.) Less.	Carducabiddu	Fl	De	Diuretic
27		* Cichorium intybus * L.	Giccoria	Le	De	Laxative
28		* Cynara cardunculus * L.	Cardureu	Le	De	Choleretic, cholagogue [34], diuretic, slightly laxative, stimulates liver functions and is useful in subacute and chronic icteric forms
29		* Helichrysum italicum * (Roth) G.Don subsp. *tyrrhenicum* (Bacch., Brullo & Giusso) Herrando, J.M.Blanco, L.Sáez Galbany	Alluevògu	WP	Inf	Diaphoretic and pectoral
30		* Matricaria chamomilla * L.	Kamomilla	Fl	De	Lenitive, decongestionant
					Inf	Digestive
31		* Senecio vulgaris * L.	Coccoininni burdu	WP	De	Astringent and diuretic
32	Boraginaceae	* Anchusa azurea * Mill.	Burraccia aresti	Le	Inf	Diaphoretic [35], expectorant
33		* Borago officinalis * L.	Pittsa carroga	Le	Inf; De	Intestinal laxative and purifying agent, diuretic
34		* Cynoglossum creticum * Mill.	Fùndu gràssu	WP	Inf	Astringent, antituberculosis [36]
35		* Echium plantagineum * L.	Erba de bòrcu	WP	De	Astringent, diaphoretic, diuretic, emollient
36		* Heliotropium europaeum * L.	Erba de soli	Fl	Inf	Astringent, vulnerary
37		* Myosotis ramosissima * Rochel	Origa de topi	WP	De	Astringent, ophthalmic, healing
38	Brassicaceae	*Capsella bursa-pastoris* (L.) Medik.	Erba de feminas	AP	Inf	Anti-hemorrhagic, regulator of menstrual flow
39		* Nasturtium officinale * R. Br.	Nasturtu	Le	De	Diuretic
					IU	Anabolic and antiscorbutic
					EU	Anti-inflammatory
40		* Sinapis arvensis * L.	Masaòccu	Se	De	Diuretic, laxative, eupeptic, rubefacient, stimulating the gastric mucosa
41		* Teesdalia coronopifolia * (J.P.Bergeret) Thell	Cauliteddu	WP	De	Stomachic, antiscorbutic
42	Cactaceae	*Opuntia ficus- indica* (L.) Mill.	Figu morisca	Cl	Co	Soothing, anti-inflammatory, against fissures and mammary inflammations
43	Caprifoliaceae	* Dipsacus ferox * Loisel	Cima de pastori	Le	De	Dermatosi desquamative, eczema, folliculitis, urticaria, psoriasis
44	Caryophyllaceae	* Stellaria media * (L.) Vill.	Erba de buddas	Le	Inf	Diaphoretic
45	Cistaceae	* Cistus creticus * L. subsp. *eriocephalus* (Viv.) Greuter & Burdet	Murdegu arrubiu	Le	Inf	Balsamic and revulsive
46	Convolvulaceae	* Convolvulus arvensis * L.	Melamida pitticca	Rt	Inf	Cathartic, drastic purgative [36], cholagogue, against skin affection
47	Crassulaceae	* Sedum * sp. pl.	Erba grassa	Le	De	Astringent, emollient, healing in skin ulcers
48		* Umbilicus horizontalis * (Guss.) DC.	Calixi de muru	Le	Inf	Diaphoretic, refreshing
49		* Umbilicus rupestris * (Salisb.) Dandy.	Capeddu de muru	Le	Inf	Diaphoretic, refreshing
					Co	Against boils, distortion, hematomas, soothing in skin inflammatory states
50	Cytinaceae	* Cytinus hypocistis * L.	Cabòne de murdegu	WP	Co	Astringent, tonic, hemostatic
51		* Cytinus ruber Fourr. * ex Fritsch.	Kobòne de murdegu	WP	Co	Astringent, tonic, hemostatic
52	Dioscoreaceae	* Dioscorea communis * (L.) Caddick & Wilkin	Agina de margiani	Rt	De	Urinary anti-inflammatory [37], diuretic, emetocathartic, hemolytic, vulnerary
53	Ericaceae	* Arbutus unedo * L.	Olidoni	WP	IU	Urinary and intestinal anti-inflammatory
54		* Erica arborea * L.	Tuvara	Le	De	Uro-genital disorders
55	Euphorbiaceae	* Euphorbia helioscopia * L.	Lua burda	Lt	IU	Strongly emetic and laxative
56	Fagaceae	* Quercus ilex * L.	Ilixi	Se	IU	Coffee substitute
57		* Quercus pubescens * Willd.	Orròli	Se	De	Against dysentery, gastralgia, eutrophic in lymphatic and tuberculous disease
58	Gentianaceae	* Blackstonia perfoliata * (L.) Huds.	Centàurea groga	WP	Inf	Biter, stomachic
59		* Centaurium maritimum * (L.) Fritsch	Brundedda	WP	Inf	Digestive, cleansing, and healing
60	Geraniaceae	* Geranium purpureum * Vill.	Erba de agullas	Le	De	Against infections and inflammation of the oral cavity
					IU	Tuberculosis
					Po	Against brush
61		* Geranium robertianum * L.	Erba de agullas	Le	De	Against infections and inflammation of the oral cavity
					IU	Tuberculosis
					Po	Against brush
62	Hypericaceae	* Hypericum perforatum * L.	Erba de S. Giuanni	Re	--	Against ulcerations and burns
				Fl	Inf	Abdominal pain, bronchial and urinary inflammation
63	Iridaceae	* Crocus sativus * L.	Zafanau	Fl	Inf	Antispasmodic [38], carminative, stimulant, emmenagogue, expectorant
64		* Limniris pseudacorus * (L.) Fuss.	Lillu grogu de arriu	Rt	De	Emetocathartic, epidermal astringent, and hemostatic
65	Juncaceae	* Luzula forsteri * (Sm.) DC.	Erba lutza	Rt	De	Against gallstones
66	Lamiaceae	* Ballota nigra * L.	Marrupiu nieddu	Le	Inf	Antispasmodic, sedative, vermifuge
67		* Clinopodium vulgare * L.		Le	De	Carminative, stimulating, emmenagogue
68		* Lavandula stoechas * L.	Abioi	Le	De	Antiseptic [39], antispasmodic, diuretic, digestive system stimulant
					Ca	Against dislocations, wounds, sores, and ulcers
69		* Marrubium vulgare * L.	Marrupiu	Fl	Inf	Tonic, intestinal purifier, weak action cardiac activity, thins the mucous secretions of the airways
70		* Melissa officinalis * L.	Ment’i àbis	WP	De	Antispasmodic [40], choleretic, stimulating and tonic
71		* Mentha pulegium * L.	Amenta	Le	Inf	Aromatic and refreshing, bechico, bronchodilator, against digestive system disorders
72		* Origanum vulgare * L.	Origanu	Le; Fr	Co	Analgesic
				Le	IU	Dental pain reliever
73		* Prunella laciniata * L.	Brunella	Le; Rt	Inf	Astringent, against respiratory and gastrointestinal diseases
74		* Salvia rosmarinus * Schleid.	Spiccu	Le;	Inf	Carminative and digestive, hepato-stimulating, antiseptic and intestinal antispasmodic, against asthma and bronchitis
75		* Salvia * sp. pl.	Salvia	Le	IU	Antiseptic, odontalgic, and against halitosis
76		* Stachys glutinosa * L.	Scova de argolas	Le	De	Cholagogue, diuretic, hepatoprotective
77		* Teucrium chamaedrys * L.	Camedriu	AP	De	Antipyretic, astringent, tonic
78	Lauraceae	* Laurus nobilis * L.	Lauru	Le	De; Inf	Antiseptic, stimulant, febrifuge, sedative colic spasms
79	Leguminosae	* Anagyris foetida * L.	Tilibba	Le	Inf	Mild laxative, emetic emmenagogue
80		* Ceratonia siliqua * L.	Karrubba		Co	Against cough and tonsillitis
				Se	De; Inf	For emollient baths
				AP	Inf	Anti-inflammatory of the throat and digestive system, astringent, antibacterial
81		* Lotus corniculatus * L.	Truvulleddu	Fl	Inf	Sedative for anxiety, insomnia, depression, and tachycardia
82		* Lupinus gussoneaus * J.Agardh.	Lupinu	Se	Inf	Vermifuge and hypoglycemic [41]
83		*Ononis spinosa* L. subsp. *antiquorum* (L.) Arcang.	Stasibois	Rt	De	Increased diuresis, decreased inflammatory processes
84	Linaceae	* Linum usitatissimum * L. subsp. *angustifolium* (Huds.) Thell.	Linu	Se	Ca	Revulsive in inflammations of the respiratory system
85	Lythraceae	* Punica granatum * L.	Arenada	Le	De	Antifungal and reduces sweating, antiviral [42,43]
86	Malvaceae	* Malva sylvestris * L.	Narbedda	Le	De	Against boils, chilblains
					IU	Bronchial renal and intestinal anti-inflammatory, suppurative lesions, fistulas
					Co	Against boils, chilblains, suppurative lesions, fistulas, and hemorrhoids
				WP	Fu	Processes inflammatory in the throat
87	Molluginaceae	* Corrigiola litoralis * L.		Le; Fl	De	Diuretic
88	Moraceae	* Ficus carica * L.	Figu	Lt	EU	Eradication wart
89	Myrtaceae	* Eucalyptus * sp. pl. Dehn.	Occallitu	Le	Co	Stimulant for external and internal use
90		* Myrtus communis * L.	Mirtu	Le	De	Balsamic, expectorant and diuretic
				Fr	IU	Digestive and aperitif
91	Oleaceae	* Olea europaea * L.	Ollastu	Le	De	In cases of biliary lithiasis
92		* Phillyrea latifolia * L.	Arrideli	Le	De	Diuretic, tonic astringent
93	Onagraceae	* Epilobium hirsutum * L.	Frori de acqua	Rt	De	Preparation of astringent mouthwashes against mouth ulcers
94	Orchidaceae	* Ophrys apifera * Hud.	Orchidea aresti	Bu	De	Anti-inflammatory gastrointestinal, against childhood diarrhea, cystitis, and nephritis
95	Papaveraceae	* Fumaria capreolata * L.	Fumària bianca	WP	De	Bitter, diaphoretic, purifying, stimulating the secretions of the digestive system
96		* Papaver rhoeas * L.	Babbaòi	Fl	Inf	Bechic, diaphoretic, broncho sedative and narcotic-sedative
97	Pinaceae	*Pinus* sp. pl.	Oppinu	Le	De	Colds and joint pains
98	Plantaginaceae	* Linaria pelisseriana * (L.) Mill.	Angulias	Fl; Le	Inf	Against angiocholitis with jaundice, intestinal atony, urinary tract disorders
99		* Plantago coronopus * L.	Erba sterria	WP	Sy; De	Astringent, blood coagulant
100		* Veronica anagallis-aquatica * L.	Murutzu aresti	WP	IU	Diuretic, antiscorbutic, purifying
101	Poaceae	*Avena barbata* Pott. Ex Link.	Enargu	AP	De	Emollient in bronchial inflammatory processes
				Le; Rt	Inf	Diuretic
102		* Cynodon dactylon * (L.) Pers.	Cannajoni	WP	De	Anti-inflammatory of the digestive tract and the uro-genital system
103		* Lolium rigidium * Gaudin	Allorgu	Le	De	Antineuralgic, astringent, sedative
104	Polygonaceae	* Polygonum aviculare * L.	Erba de zentu nùus	AP	Inf	Astringent in case of internal bleeding, mild laxative, and blood purifier
105		* Rumex crispus * L.	Melagra	Le	De	Astringent for boils, abscesses, myalgias, sprains
106	Primulaceae	* Lysimachia arvensis * (L.)	Erba de puddas	Le	De	Expectorant [44], diaphoretic, diuretic, cholagogue
					Ca	Against sores, ulcers, skin affection [11]
107		* Lysimachia foemina * (Mill.) U.Manns & Anderb.	Erba de puddas	Le	De	Expectorant, diaphoretic, diuretic, cholagogue
					Co	Against sores, ulcers, skin affections
108	Pteridaceae	* Adiantum capillus-veneris * L.	Fartsia	AP	De	Expectorant, emollient, adjuvant bronchial affections
109	Ranunculaceae	* Anemone hortensis * L.	Anemoni	Le	Inf	Rubefacient vesicatory, against skin rashes, joint rheumatism, sciatica
110		* Clematis flammula * L.	Bintisillu	Le	Inf	Rubefacient, vesicatory, against rheumatism, gout
111		* Clematis vitalba * L.	Pipiringiu	Rt; AP	TU	Analgesic for diseased teeth or horns of animals
112		* Ficaria verna * Huds.	Landiri de terra	Rt	De	Analgesic, anti-hemorrhoidal, hemostatic
113		* Ranunculus macrophyllus * Desf.	Cadedda	Le	Ca	Revulsive and rubefacient against rheumatic forms, in arthrosis and sciatica
114	Resedaceae	* Reseda luteola * L.	Erba de gallu	Le	Inf	Diaphoretic, diuretic, stomachic
115	Rhamnaceae	* Ziziphus jujuba * Mill.	Isaba	Fr	De	Respiratory tract anti-inflammatory
116	Rosaceae	* Agrimonia eupatoria * L.	Erba mela	Le	De	Astringent, mouthwash, against inflammation of the digestive system and against liver and kidney disorders
117		* Crataegus laevigata * (Poir.) DC.	Travigu	Le; Fl; Fr	De	Vasodilator, hypotensive, antiarrhythmic, sedative [45]
118		* Crataegus monogyna * Jacq.	Soarviu	Fl	De	Against hypertension, cardiac, cardiac neurosis and angina pectoris, antispasmodic ad against anxiety and insomnia
				Ba	De	febrifuge
119		* Potentilla reptans * L.	Erba de cincu follas	Le	Inf	Astringent, stomachic, antiscorbutic, febrifuge
120		* Poterium sanguisorba * L.	Pimpinella	WP	Inf	Astringent, and against acute and chronic intestinal diseases
121		* Prunus spinosa * L.	Prunizedda	Fl	De	Laxative and diuretic, intestinal astringent
				Ba	Inf	Intestinal astringent
122		* Rosa canina * L.	Arrosa burda	Le	Inf	Analgesic
				Fl	De	Astringent, tonic [46], ophthalmic
				Fr	Inf	Against urinary tract diseases, and in cases of diabetes
123		* Rubus ulmifolius * Schott.	Arrù	Fr	De	Refreshing and light laxative
				Le	De	Mouthwash preparations for astringent and anti-inflammatory gargling
124	Rubiaceae	* Galium aparine * L.	Appiciga	WP	Inf	Antispasmodic, slightly diuretic, astringent, against digestive system disorders and skin disease
125	Rutaceae	* Ruta chalepensis * L.	Arruda	Le	IU; Oil	Against odontalgia, oral cavity infections
126	Scrophulariaceae	* Scrophularia trifoliata * L.	Suisùi	Le	Inf	Emetic, purgative [47], against the manifestations of Grave’s disease and related cardiac disorders
127		* Verbascum creticum * (L.) Kuntze	Cadumbu	Le	De	Emollient, decongestant, anti-inflammatory of the intestinal mucosa
128	Smilacaceae	* Smilax aspera * L.	Tittione	Le	De	Diaphoretic [25,48]
129	Solanaceae	* Hyoscyamus niger * L.	Nasturru	Le; Se	De	Against trigeminal neuralgia, attenuation of senile tremor in Parkinson’s disease, antispasmodic, local anesthetic and analgesic [49]
130		* Solanum nigrum * L.	Margaridraza	Le	Inf	Anti-inflammatory [50], emetocathartic, spasmolytic, against skin affections and analgesic
131	Tamaricaceae	* Tamarix africana * Poir.	Tramattsu	Ba	De	Astringent, diaphoretic
132	Thymelaeaceae	* Daphne gnidium * L.	Truiscu	Le	Inf	Diaphoretic, emetocathartic, rubefacient, vesicatory [51]
133		* Thymelaea hirsuta * (L.) Endl.	Scova de forru	Fl	Inf	Rhinitis and asthma [52]
134	Ulmaceae	* Ulmus minor * Mill.	Ulumu	Le; Ba	De	Intestinal astringent [24]
135	Urticaceae	* Urtica dioica * L.	Occiau	Le	De; Inf	Astringent [53], hemostatic, hypoglycemic, depurative, diuretic, against headaches and digestive and heart problems
					Co	Pain reliever
136		* Urtica pilulifera * L.	Occiau femina	Le	De	Astringent, hemostatic, hypoglycemic, urtication for revulsive purposes in cases of paralysis and joint rheumatism
137	Violaceae	* Viola alba * Besser subsp. *dehnhardtii* (Ten.) W.Becker	Violedda	Le	De	Emollient, expectorant

Part Used: AP, aerial parts; Ba, barks; Bu, bulb; Cas, cataphylls; Cl, cladodes; Fl, flowers; Fr, fruits; Le, leaves; Lt, latex; Re, resin; Rt, root; Se, seeds; UP, underground part; WP, whole plant. Preparation: Ca, cataplasm; Co, compress; De, decoction; DI, direct ingestion; EU, external use; Inf, infusion; IU, internal use; Oil; Po, poultice; Sy, syrup; Fu, fumigation; TU, topic use.

Life Forms analysis of the medicinal plants (Table 2) revealed that the majority were hemicryptophytes with 32%, followed by phanerophytes (27%), therophytes (24%), geophytes (13%), while the least represented form was the chamaephytes, with 4%. Regarding the Chorotype, (Table 2) unsurprisingly, 54.4% of the species belong to the Mediterranean chorotype, while approximately 30% of medicinal plants are equally divided into European, Cosmopolitan, and Subcosmopolitan chorological types. The least represented are, respectively, Tropical and Subtropical (4.4%), Circumboreal (2.2%), Asian (2.2%), and Neotropical (1.5%) chorologic types, the latter consisting of *Eucalyptus* sp. pl. Dehn. from Australia and *Opuntia ficus-indica* (L.) Mill from Mexico. Finally, only 2.9% consists of endemic plants.

**Figure 2:**
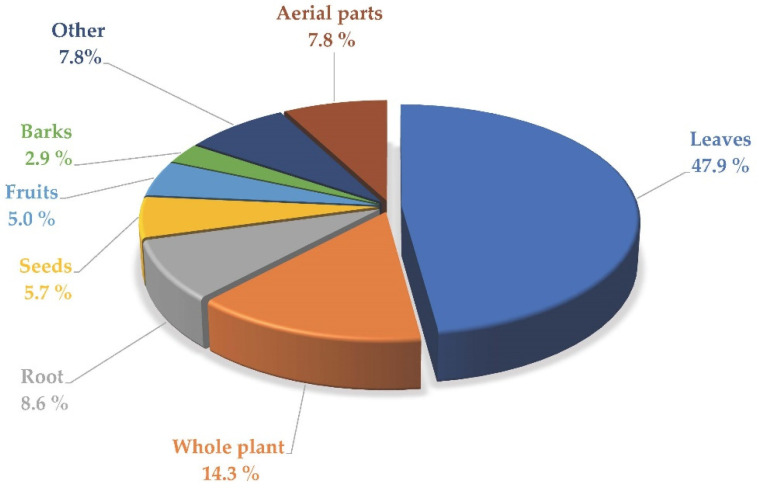
Part of the plant used.

The data collected provided us with a complete overview of the part of the plant used, reported in Figure 2. In the vast majority of the medicinal plants analyzed, the herbal drugs were represented by the *leaves* (47.9%), while a minority is constituted by *fruits* (5%), *seeds* (5.7 %), and *barks* (2.9 %).

**Figure 3:**
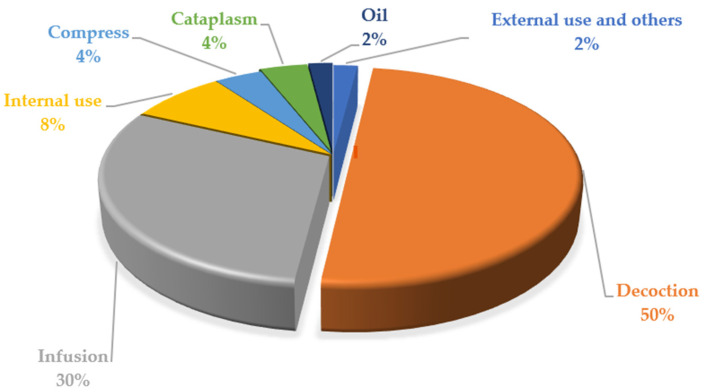
Preparations of the herbal drugs (percentage).

Subsequently, we investigated the preparations of the herbal drug (Figure 3). According to the participants, the main preparations were *decoction* (50%) and *infusion* (30%), followed by internal use (8%), *compress* and *cataplasm* (4%), and lastly, external use and others (2%).

**Figure 4:**
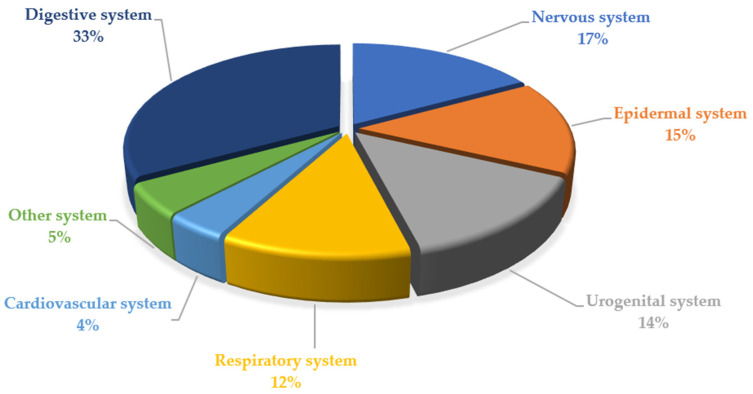
Folk therapeutical uses (percentage).

Finally, we collected and organized the reported effects of medicinal plants (Table 1), deepened with the help of a local medical doctor during the interview. Overall, these preparations were used in Marmilla’s folk medicine to treat pathologies of the digestive system, 33%, while the other principal uses regarded nervous, epidermal, and urogenital systems, 17%, 15%, and 14%, respectively (Figure 4).

**Figure 5:**
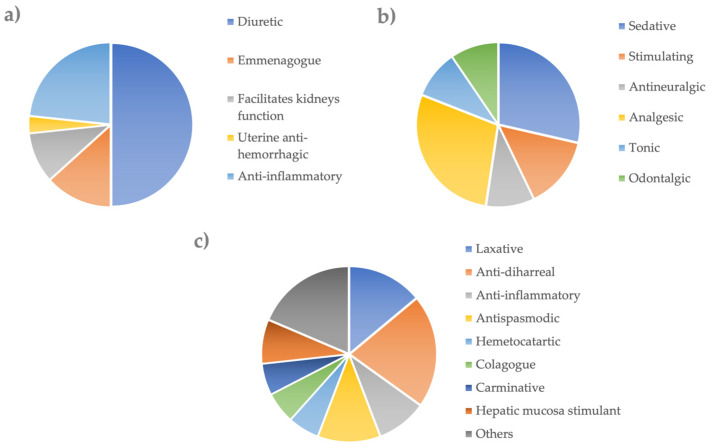
Folk therapeutical uses. The effects at the Urogenital (**a**), Nervous (**b**), and Digestive system (**c**).

As regards the digestive apparatus, the species used reported anti-diarrheal, laxative, and antispasmodic properties (Figure 5). The effects at the uro-genital apparatus level span from diuretic, anti-inflammatory, and emmenagogue effects (Figure 5). Finally, at the nervous system level, the properties concern analgesic, sedative, stimulating, and antineuralgic effects (Figure 5).

**Figure 6:**
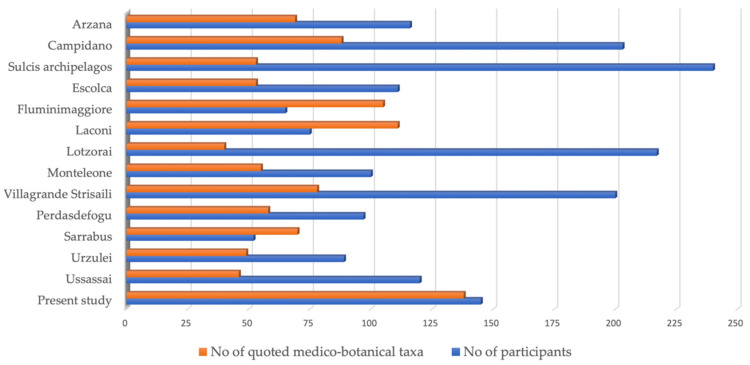
Number of interviewed participants and quoted medico-botanical taxa in the present study, compared with those from other ethnobotanical studies conducted in Sardinia.

Compared to previous ethnobotanical research conducted on the island [20,21,22,23,24,25,26,27], an interesting finding relates to the number of plants provided compared to the people interviewed (Figure 6).

For the same number of participants, a higher number of cited taxa may indicate good conservation of TBK, while a low number conversely indicates erosion. This was observed in Sardinia during the ethnobotanical survey of the Ligurian minority, the Tabarkin community in the Sulcis archipelago [22].

With the aim of representing these concepts numerically, we compared the R ratio between participants and taxa (I/T). Although this parameter is relative, it can be used to compare ethno-botanical studies confined to homogeneous territories (influenced by the same phenomena over time). For this, the R ratio was used for the comparison between the ethnobotanical studies of the island subregions and the single villages, such as Sarrabus, Campidano, Sulcis archipelago, and Marmilla, discussed in the present study.

This ratio, here called R, can provide a quick and useful parameter to verify TBK erosion in communities: for low R values, around zero and one, the number of species exceeds the number of participants, suggesting that traditions are well established in the area. Conversely, high R values, far above one, may suggest an onset of cultural erosion.

The present study has R values just above 1 (R = 1.05), a value that does not indicate erosion, also evidenced by the highest number of taxa among the studies under review, which may indicate a good conservation of TBK.

In general, the value of R can be used quickly and immediately for data comparison, however, it should be considered that it provides an indication that needs to be proven by knowledge of the phenomena occurring in the area.

## 3. Discussion

The present work provides extensive documentation of the medicinal plants used by the community of Marmilla, obtained through a blending arrangement of different disciplinary competencies. The participants here played the leading role, transmitting their local cultural knowledge. In addition, they visually recognized fresh plants or dried specimens, helping with plant identification and with translation from the vernacular to the scientific name. Subsequently, with the aid of the local doctor, the reported effects were identified in medical and diagnostic terms. The vernacular name was later documented and shown in the table since it is fundamental not only for identifying the plant by the community but also because it often represents the description of the plant itself, both from the morphological point of view and from the therapeutic action described. As reported in the results (Figure 3), leaves were the most used herbal drug. Interestingly, the bibliographic analysis revealed the use of plants containing toxic substances. In some instances, these substances are avoided by carefully choosing the herbal drug, such as the toxic alkaloids in the roots of *Prospero autumnale* (L.) Speta or the poisonous berries of *Solanum nigrum* L., rich in solasodine, a steroidal alkaloid [54], when un-ripe. In both these cases, the local population uses the leaves as a herbal drug, in which the toxic principle is absent. Again, the species *Clematis vitalba* L. contains saponins and alkaloids such as anemonine and protoanemonine, which are caustic and irritating. In this case [55], the local population refers to the use of roots and branches, softening the effect through topical use as an analgesic. A further example, *Lysimachia arvensis* (L.) U. Manns & Anderb. contains saponins, flavonoids, and tannins present in every part but mainly concentrated in the seeds, which can even cause severe phenomena of gastric irritations and contact dermatitis [56]. In this case, local people use the leaves as a poultice and as a decoction.

While often the toxic principle is cleverly avoided, in other cases, it is exploited just for this reason. Here, we found effects concerning the digestive system, as the instance of the latex of *Euphorbia helioscopia* L., used as an emetic and laxative [57], and the infusion obtained from the leaves of *Daphne gnidium* L., containing mezerein and daphnine, with vesicatory, rubefacient and purgative action [58]. Also interesting is the use of toxic principles which have an effect at the nervous system level, as reported for *Hyoscyamus niger* L., toxic in all its parts if ingested, causing convulsions, respiratory difficulties, and death [59]. Nevertheless, the local community uses it as a decoction in order to relieve symptoms of trigeminal neuralgia, Parkinson’s disease, and senile tremor. Moreover, it has local anesthetic, antispasmodic, and analgesic activities. The effects are recognized and used at the pharmaceutical level to prepare antispasmodic and antineuralgic products acting on smooth muscles. Still, *Hedera helix* L. is used by the local community to treat neuritis and neuralgia, even though it is reported to be toxic if ingested as it contains triterpene saponins and alkaloids [60].

In summary, the common use of toxic plants in folk medicine may suggest local knowledge of the plant properties, developed through hundreds of years of trial and error, constituting a primitive clinical trial. However, it also highlights possible concerns regarding the safety and security of herbal drugs.

In respect of the reported effects, the targeted use against specific pathologies can also be linked to the ethnic characteristics of the population. As previously mentioned, the Sardinian human population has been isolated for a long time, modifying its genetic structure [61]. Furthermore, several studies have revealed the significant presence of inflammatory and autoimmune diseases, defining the island as an autoimmune hotspot [62] and the subject of numerous studies [63,64]. Notably, 19 plants are reported to have anti-inflammatory properties, while eight plants are reported to have specific antirheumatic properties. As regards the latter, Ranunculaceae appear particularly noteworthy, as three plant species out of eight belong to this family and have been found to exhibit antirheumatic activity (*Anemone hortensis* L., *Clematis flammula* L., and *Ranunculus macrophyllus* Desf.). As a result, it can be assumed that the population’s specific traits have therefore conditioned the relationship between the people and the usage of medicinal plants present in the territory.

Another aspect of this study is that nearly 60% of the plants locally used for medicinal aims are Mediterranean endemic taxa, of which four (2.9%) are narrow endemic plants, with a restricted range and extremely valuable as a source of new bioactive molecules [47]. Therefore, among the four narrow endemic plants that emerged, we conducted a literature research focusing on both similar ethnopharmacobotanical uses and pharmacological activity.

*Dipsacus ferox* Loisel has reported beneficial effects at the epidermic level, being used against desquamative dermatoses, eczema, folliculitis, urticaria, and psoriasis. A comparative literature review showed it to be used in different parts of the Island for food purposes [65,66], and by the Ogliastra community (Nuoro Province) as an antieczemic, confirming the benefits against epidermic inflammatory diseases [67].

From a chemical and pharmacological point of view, few studies have been found related to *D. ferox*, characterized by the presence of iridoids, distinctive compounds in Dipsacaceae [68]. More can be said about the pharmacological activity of *Dipsaucus* genus, which is reported to have anti-aging, anti-inflammatory, anti-bone fracture, hepatoprotective, and anti-myocardial infarction activity. Moreover, studies show its activity against inflammation-based diseases such as osteoarthritis [69,70,71].

*Stachys glutinosa* L. is employed by the Marmilla community as a cholagogue, diuretic, and hepatoprotective. Various and different ethnobotanical uses have also been found within the Island, from the simplest use against colds to antiseptic, antispasmodic, and sedative applications [20,67,72].

Essential oils and extracts of *S. glutinosa* have been chemically well characterized, while in vitro tests have shown mild antiproliferative activity against cellular tumor lines [73,74,75]. Further studies have then shown an affinity for opioid receptors, and good bacteriostatic activity against certain types of bacteria and fungi [76].

In the present study, *Scrophularia trifoliata* L. is reported to have purgative and emetic effects, and to be used against Grave’s disease and related heart disorders. Interestingly, it was found that this endemic plant is used by different local populations in Sardinia to treat various diseases, often related to inflammatory or autoimmune conditions, such as Grave’s disease in Marmilla, antirheumatic activity in Ussassai, Urzulei and Villagrande Strisaili (Nuoro Province), Escolca (South-Sardinia Province), and anti-inflammatory activity in Aggius (Sassari Province) [20,21,25,26]. Its extracts have shown anti-HIV activity in vitro [77,78,79], while the genus *Scrophularia* has antioxidant and anti-inflammatory activity [78,79,80]. Notably, several studies reported how *S. striata* Boiss. inhibits the production of NO [81] and pro-inflammatory cytokines [82]. When produced in excess, these molecules are associated with several diseases such as chronic inflammatory, septic shock, and autoimmune diseases. Due to its immunomodulatory activity, its potential effects against COVID-19 inflammation have been highlighted [83].

The fourth endemic plant, *Helichrysum microphyllum* (Willd.) Cambess. subsp. *tyrrhenicum* (Bacch., Brullo & Giusso) Herrando, J.M.Blanco, L.Sáez Galbany, is reported to have diaphoretic and expectorant action in the present study. In contrast, ethnobotanical studies on the whole of Sardinia show anti-allergic effects, against skin diseases and alopecia, bronchitis, laryngitis, tracheitis, cough sedative, antineuralgic and antirheumatic [20,21,22,25,84]. *H. italicum* subsp. *tyrrhenicum* is both the most studied endemism and also the most chemically variable [85,86]. Indeed, it has been shown that some compounds are increased in winter with low temperatures, such as nerolidol, in contrast, italicene, bergamotene, nerol and curcumene, are positively affected by high temperatures and therefore present during spring and summer [87]. This endemism also shows antiviral activity against HIV and strong antimicrobial activity against multidrug-resistant *Staphylococcus aureus* isolates, among others, and anti-fungal activity against *Candida* spp. [88,89,90,91].

In general, ethnobotanical uses appeared to be confirmed by tested pharmacological properties. Notwithstanding, there are still few studies for some plants, especially endemic ones, which due to their potential in terms of chemical biodiversity, deserve to be further characterized.

Another element to consider in this regard is the preservation of biodiversity. The uncontrolled collection of wild plants for medicinal purposes might be harmful to the conservation of local populations. The medicinal plants here obtained have been compared both to the European and Italian red lists [92,93] and to the up-to-date IUCN online source (https://www.iucnredlist.org), and from this comparison, 71 Least Concern (LC), 4 Data Deficient (DD), 4 Near Threatened (NT), and 1 Vulnerable (VU) species were found.

The Red List presents critical indicators of biodiversity status, highlighting the threatened species. Nevertheless, classification is still an ongoing process that requires the collaborative efforts of researchers, in fact, only a fraction of known species is currently categorized.

In the present research, 80 plants out of 137 were already assessed with the IUCN criteria. Particularly, here emerged the need for the protection of the Near Threatened plants, *Helosciadium crassipes* W.D.J.Koch ex Rchb., *Marrubium vulgare* L., *Oenanthe fistulosa* L., and *Scrophularia trifoliata* L. Moreover, further precautions should be used when the medicinal effect is found at the level of bulbs and roots. In this case, harvesting the plant may pose a risk to the plant’s survival. The orchid *Ophrys apifera* Huds. is a perfect case in point.

In conclusion, on the one hand, the preservation of TBK should be monitored for the conservation of biodiversity, particularly in hotspots of global biodiversity like the Mediterranean basin and its large islands, through raising public awareness about endangered species and avoiding their uncontrolled collection. On the other hand, it is possible that strong conservation stems from a combination of demographic and geomorphological characteristics of the territory. From the demographic point of view, Sardinia has been recognized as one of the four Blue Zones globally, characterized by extreme longevity. Specifically, Marmilla is adjacent to the focus territory of the Sardinian Restricted Blue zone, therefore, it is conceivable that the population’s high seniority, combined with these historical, geomorphological, and cultural characteristics of sub-regions, has led to good conservation of TBK over time. From this perspective, it is even more interesting to discover the natural remedies, which, together with diet and life habits, contribute to maintaining such a long-lived community.

## 4. Material and Methods

### 4.1. Historical and Ethnographic Background

The territory of Marmilla plays a principal role in the present research, and the ethnobotanical interviews revealed that medicinal plants employed in the local tradition are commonly collected still today.

**Figure 7:**
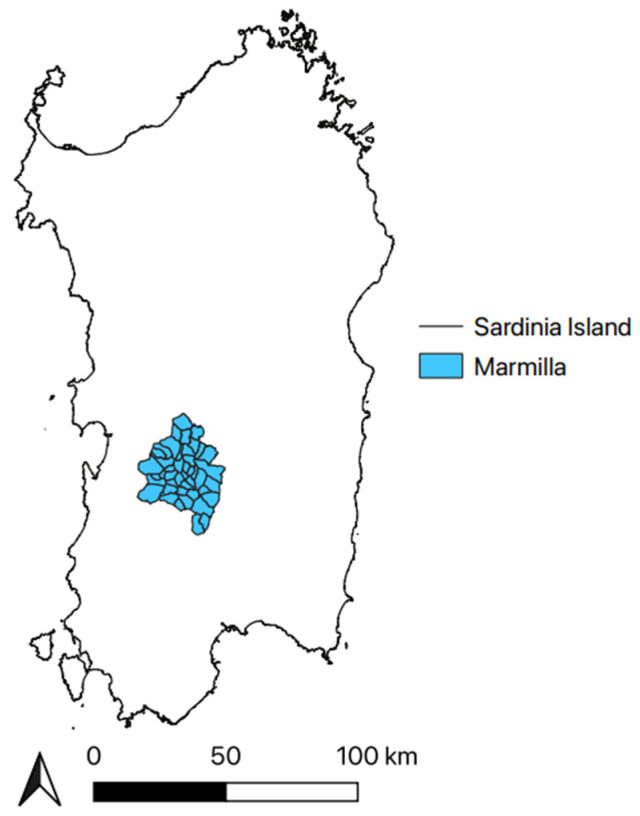
Study Area.

Marmilla is a historical subregion of central-southern Sardinia, located between 39°47′ and 39° 30′ North latitude and between 8°47′ and 9°12′ East longitude (Figure 7), presenting a territorial extension of 415 sqKm [94]. The Flumini Mannu river morphologically defines its borders to the south-east, Giara of Gesturi to the north, and Monte Arci to the west. It consists of two sub-areas, called “Alta Marmilla” and “Bassa Marmilla”. The first one belongs to the province of Oristano and extends between two natural monuments: the Giara plateau and the Monte Arci, a volcanic massif, rich in obsidian, volcanic glass used by prehistoric populations for the production of tools. It borders to the south with the second sub-area, “Bassa Marmilla”, which belongs to the province of Medio Campidano.

From an historical point of view, there is evidence that dates back to the Middle Bronze Age (ca. 1600–1500 BCE), demonstrating that Marmilla has been inhabited since ancient times. Indeed, the territory is characterized by several ‘Nuraghes’, typical buildings of the so-called Nuragic civilization, including 139 different Nuragic sites. Among others, the Nuragic site of Barumini deserves a particular mention, representing the leading Nuragic site in Sardinia and being included in the United Nations Educational, Scientific and Cultural Organization’s [UNESCO] list of World Heritage Sites in 1997. Further historical evidence is linked to the colonization in the first century CE by the Romans and in the Middle Age by the Kingdom of Arborea, with the construction of Marmilla’s Castle, an area of strategic and military importance. The territory is characterized by a predominantly agricultural economy and a permanent resident population. The present work focused on five municipalities, Furtei, Gesturi, Segariu, Tuili, and Villamar, small villages with a population between 1000 and 2500 inhabitants, with an overall average of 1500 inhabitants. Similarly, to the whole Marmilla territory, these villages are affected by high emigration rates and older demographic profiles. In addition, the weakness of the economic structure is resulting in progressive depopulation [94].

### 4.2. Environmental Background

Sardinia is located in the center of the western Mediterranean basin. The territory shows remarkable differences from geological and morphological points of view, dividing the island into sectors. The result of the ancient geological history of the island is clearly visible in the central-eastern part of the island, in which there are the most ancient rocks of the Paleozoic age, from the Ercinic orogeny. This orogenic phase has led to metamorphism and magmatism phenomena, forming the Sardinian basement. Subsequently, Sardinia drifted away from southern France with the Alpine orogeny, separating permanently from the European block, and settling in the center of the Mediterranean basin. The central-western part extends the Sardinian-Campidanese graben, a sedimentation basin consisting primarily of marine sedimentary rocks interspersed with volcanites, resulting from the erosion of the neighboring areas. These ancient geological processes have shaped the island, which, from a morphological point of view, has a reduced mountainous component due to the consequent erosion. In contrast, the smoothened hills confer more than 50% hilly components, with altimetry between 200 and 700 m [95]. This heterogeneity, especially in the inner territories, limited the communities’ exchanges. The climate is the Mediterranean Pluviseasonal Oceanic type, with evident seasonality, consisting of hot and dry summer and mild winter. However, the union of different factors (among others, temperature, continentality, and precipitation) results in highly diverse isobioclimates, with a total of 43 [95]. This variability is reflected in a considerable diversity of environments that have contributed to the development of high endemism over the centuries. At present, Sardinia’s native flora is constituted of 2300–2500 species and subspecies [96,97] of which 15% are considered endemic to the island [31]. Considering vegetational, physiographic, bioclimatic, and biogeographical factors, the latest studies divided Sardinia into 23 main vegetation series. The main ones are the woodland formations, distinguished in oaks (*Quercus coccifera* L., *Quercus ilex* L., *Quercus suber* L., *Quercus* gr. *pubescens* Willd.), junipers (*Juniperus communis* L., *Juniperus macrocarpa* Sm., *Juniperus oxycedrus* L., *Juniperus turbinata* Guss.), wild olive (*Olea europea* L.,), pines (*Pinus halapensis* Mill., *Pinus pinaster* Aiton, *Pinus pinea* L.) and other tree species covering smaller areas (*Acer monspessulanum* L., *Castanea sativa* Mill., *Ilex aquifolium* L., *Laurus nobilis* L., *Taxus baccata* L.) [98]. Another typical vegetation type is the Mediterranean maquis, with evergreen formations exceeding 4 m [99]. The main exponents are *Arbutus unedo* L., *Chamaerops humilis* L., *Myrtus communis* L., *Pistacia lentiscus* L., *Rhamnus alaternus* L., *Salvia rosmarinus* Schleid., *Smilax aspera* L. and many species of the genera *Cistus* L. and *Genista* L. Finally, the garrigue, shrubby vegetation formed by low plants that grow in an isolated way and can be found up to 1400 m of altitude. This vegetation is represented mainly by *Helichrysum italicum* (Roth) G.Don subsp. *tyrrhenicum* (Bacch., Brullo & Giusso) Herrando, J.M.Blanco, L.Sáez Galbany, *Thymus herba-barona* Loisel., *Cytisus spinosus* (L.) Lam., *Genista corsica* (Loisel.) DC., *Teucrium marum* L., *Lavandula stoechas* L. subsp. *stoechas*, *Santolina corsica* Jord. & Fourr and *Santolina insularis* (Gennari ex Fiori) Arrigoni.

### 4.3. Data Collection

Ethnobotanical research was carried out in the municipalities of Furtei, Gesturi, Segariu, Tuili, and Villamar. The survey occurred from February 2017 to March 2018, and the participants’ sample was selected purposively over 65 years of age. It involved a final sample of 145 people, 94 men and 51 women (64.8% men and 35.2% women). Semi-structured interviews with pre-formulated questions were conducted, according to the survey method used by [100,101] through guide interviews aimed at compiling a pre-formulated form. The surveys were performed both in Italian and vernacular languages. The subsequent systematic classification of the species was carried out following the guidelines of Flora d’Italia [102]. For the updated classification of the plant families, we followed the Bartolucci et al. classifications [96]. Regarding the survey field, the acquired data are processed and compared with the plant samples used (whole plant or parts of it).

The plant matrices were stored with the most appropriate methodology according to the plant, if complete, portioned, chopped, fresh or dried, packaged or otherwise. The difficulty at this stage lies in the degree of botanical knowledge of the people interviewed in identifying plant species and the need for translation from the vernacular name to the scientific name corresponding to the plant matrix indicated. As for the dried plant drugs used in traditional medicine, conservation techniques relevant to classical pharmacognosy were used. In the laboratory, the samples were identified following standard phytognosy techniques, when possible. Where it was not possible, for example, because of mixtures of different plant drugs, we proceeded to the recognition of drugs and their subsequent identification by analysis of macroscopic (shape, size, etc.) and microscopic (absence, presence of starch, etc.) characteristics, sensory (bitter, sweet, aromatic, etc.) and tests with chemical solvents and reagents. For the identification, we also used the consultation of relevant literature and the Cagliari Herbarium (CAG) of the University of Cagliari. Finally, for a more precise diagnosis of the diseases reported by the participants, we used the cooperation of local medical doctors and their valuable knowledge about the health of their patients.

### 4.4. Data Analysis

The data collected during the interviews were processed on Microsoft Excel and organized in Table 1, divided into plant species, vernacular name, traditional use, herbal drug, and preparation.

The Excel spreadsheet was further processed, grouped by homogenous categories (family, preparation, endemism, etc.), expressed in percentage, and reported in the figures.

The ratio between the No. of participants and No. of emerged medico-botanical taxa in the present article was calculated. Then, it was compared with the other ethnobotanical studies in Sardinia and reported in Figure 6.

## 5. Conclusions

This paper provides extensive documentation of the ethnobotanical culture of the Marmilla subregion. The geographic and cultural isolation characterizes Sardinia and its subregions, providing its communities with unique cultural and social peculiarities. In addition, numerous studies report how isolation also shaped the population genetically. Therefore, it is even more interesting to understand the complex relationships between the population and Sardinia’s rich floristic biodiversity resulting from centuries of trial and error.

The study adds a piece to Sardinian and Mediterranean ethnobotany that is of increasing relevance given the rapid decline of folk traditions. TBK preserved in the older generations is at risk of disappearing at an accelerated pace due to the recent global coronavirus outbreak.

In addition to its fundamental role in documenting and codifying the cultural heritage of the Marmilla Subregion, the present work may also provide new targets for phytochemical and phytotherapeutic research.

## Figures and Tables

**Table 2:** Life Forms and Chorological Types.

n°	Family	Taxa	L.F.	Chorological Types
1	Adoxaceae	*Sambucus nigra* L.	*P caesp*	*Europ.-Caucas.*
2	Amaranthaceae	*Chenopodium vulvaria* L.	*T scap*	*Europ.*
3	Amaryllidaceae	*Narcissus tazetta* L.	*G bulb*	*Steno-Medit.*
4	Anacardiaceae	*Pistacia lentiscus* L.	*P caesp (P scap)*	*S-Medit.–Macarones.*
5	Apiaceae	*Bupleurum fruticosum* L.	*NP*	*Steno-Medit.*
6		*Daucus carota* L.	*H bienn (T scap)*	*Subcosmop.*
7		*Eryngium campestre* L.	*H scap*	*Euri-Medit.*
8		*Ferula communis* L.	*H scap*	*S-Medit (Euri-)*
9		*Foeniculum vulgare* Mill.	*H bienn/H scap*	*Euri-Medit.*
10		*Helosciadium crassipes* W.D.J.Koch ex Rchb.	*H rept/ I rad*	*Steno-Medit.*
11		*Oenanthe fistulosa* L.	*H scap*	*Eurasiat.*
12		*Scandix pecten-veneris* L.	*T scap*	*Subcosmop.*
13		*Thapsia garganica* L.	*H scap*	*Steno-Medit.*
14	Apocynaceae	*Nerium oleander* L.	*P caesp (P scap)*	*S-Medit.*
15	Araliaceae	*Hedera helix* L.	*P lian*	*Submedit.–Subatl.*
16	Asparagaceae	*Muscari comosum* (L.) Mill.	*G bulb*	*Euri-Medit.*
17		*Prospero autumnale* (L.) Speta	*G bulb*	*Euri-Medit.*
18		*Ruscus aculeatus* L.	*G rhiz/Ch frut*	*Euri-Medit.*
19	Aspleniaceae	*Asplenium onopteris* L.	*H ros*	*Steno-Medit.-Macarones*
20		*Asplenium trichomanes* L.	*H ros*	*Cosmop.-Temp*
21	Asphodelaceae	*Asphodelus ramosus* L.	*G tub*	*Steno-Medit.*
22	Asteraceae	*Achillea millefolium* L.	*H scap*	*Euro-Siber.*
23		*Artemisia arborescens* (Vaill.) L.	*NP/P caesp*	*S-Medit.*
24		*Bellis perennis* L.	*H ros*	*Circumbor.*
25		*Calendula arvensis* (Vaill.) L.	*T scap (H bienn)*	*Euri-Medit.*
26		*Carlina gummifera* (L.) Less.	*H ros*	*S-Medit.*
27		*Cichorium intybus* L.	*H scap*	*Cosmop.*
28		*Cynara cardunculus* L.	*H scap*	*Steno-Medit.*
29		*Helichrysum italicum* (Roth) G.Don subsp. *tyrrhenicum* (Bacch., Brullo & Giusso) Herrando, J.M.Blanco, L.Sáez Galbany	*Ch suffr*	*Endemism*
30		*Matricaria chamomilla* L.	*T scap*	*Subcosmop.*
31		*Senecio vulgaris* L.	*T scap*	*Cosmop.*
32	Boraginaceae	*Anchusa azurea* Mill.	*H scap*	*Euri-Medit.*
33		*Borago officinalis* L.	*T scap*	*Euri-Medit.*
34		*Cynoglossum creticum* Mill.	*H bienn*	*Euri-Medit.*
35		*Echium plantagineum* L.	*T scap/H bienn*	*Euri-Medit.*
36		*Heliotropium europaeum* L.	*T scap*	*Euri-Medit.–Turan.*
37		*Myosotis ramosissima* Rochel	*T scap*	*Europ.–W-Asiat.*
38	Brassicaceae	*Capsella bursa-pastoris* (L.) Medik.	*H bienn*	*Cosmop.(sinantrop.)*
39		*Nasturtium officinale* R.Br.	*H scap*	*Cosmop.*
40		*Sinapis arvensis* L.	*T scap*	*Steno-Medit.*
41		*Teesdalia coronopifolia* (J.P.Bergeret) Thell	*T scap*	*Euri-Medit*
42	Cactaceae	*Opuntia ficus- indica* (L.) Mill.	*P succ*	*Messico (Neotropic.).*
43	Caprifoliaceae	*Dipsacus ferox* Loisel	*H bienn*	*Endemism*
44	Caryophyllaceae	*Stellaria media* (L.) Vill.	*T rept/ H bienn*	*Cosmopol.*
45	Cistaceae	*Cistus creticus* L. subsp. *eriocephalus* (Viv.) Greuter & Burdet	*NP*	*Steno-Medit.*
46	Convolvulaceae	*Convolvulus arvensis* L.	*G rhiz*	*Cosmop.*
47	Crassulaceae	*Sedum* sp. pl.		
48		*Umbilicus horizontalis* (Guss.) DC.	*G bulb*	*Steno-Medit*
49		*Umbilicus rupestris* (Salisb.) Dandy.	*G bulb*	*Steno-Medit*
50	Cytinaceae	*Cytinus hypocistis* (L.) L.	*G rad*	*Medit.-Macarones.*
51		*Cytinus ruber* Fourr. ex Fritsch	*G rad*	*W-Medit*
52	Dioscoreaceae	*Dioscorea communis* (L.) Caddick & Wilkin	*G rad*	*Euri-Medit.*
53	Ericaceae	*Arbutus unedo* L.	*P caesp (P scap)*	*Steno-Medit.*
54		*Erica arborea* L.	*P caesp (NP)*	*Steno-Medit.-Atlant.*
55	Euphorbiaceae	*Euphorbia helioscopia* L.	*T scap*	*Cosmopol.*
56	Fagaceae	*Quercus ilex* L.	*P scap (P caesp.)*	*Steno–Medit.*
57		*Quercus pubescens* Willd.	*P scap*	*Europ.-Subpontica)*
58	Gentianaceae	*Blackstonia perfoliata* (L.) Huds.	*T scap*	*Euri-Medit.*
59		*Centaurium maritimum* (L.) Fritsch	*T scap*	*Steno-Medit.*
60	Geraniaceae	*Geranium purpureum* Vill.	*T scap*	*Euri-Medit.*
61		*Geranium robertianum* L.	*T scap/ H bienn*	*Subcosmop.*
62	Hypericaceae	*Hypericum perforatum* L.	*H scap*	*Subcosmop.*
63	Iridaceae	*Crocus sativus* L.	*G bulb*	*W-Asiat.*
64		*Limniris pseudacorus* (L.) Fuss.	*G rhiz*	*Eurasiat. Temp.*
65	Juncaceae	*Luzula forsteri* (Sm.) DC.	*H caesp*	*Euri-Medit.*
66	Lamiaceae	*Ballota nigra* L.	*H scap*	*Euri-Medit.*
67		*Clinopodium vulgare* L.	*H scap*	*Circumbor.*
68		*Lavandula stoechas* L.	*NP*	*Steno-Medit.*
69		*Marrubium vulgare* L.	*H scap*	*Subcosmop.*
70		*Melissa officinalis* L.	*H scap*	*Euri-Medit.*
71		*Mentha pulegium* L.	*H scap*	*Subcosmop.*
72		*Origanum vulgare* L.	*H scap*	*Eurasiat.*
73		*Prunella laciniata* (L.) L.	*H scap*	*Euri-Medit*
74		*Salvia rosmarinus* Schleid.	*NP*	*Steno-Medit.*
75		*Salvia* sp. pl.	*---*	*---*
76		*Stachys glutinosa* L.	*Ch frut*	*Endemism*
77		*Teucrium chamaedrys* L.	*Ch suff*	*Euri-Medit*
78	Lauraceae	*Laurus nobilis* L.	*P caesp (P scap)*	*Steno-Medit.*
79	Leguminosae	*Anagyris foetida* L.	*P caesp*	*S. Medit.*
80		*Ceratonia siliqua* L.	*P caesp/ P scap*	*S.Medit.*
81		*Lotus corniculatus* L.	*H scap*	*Cosmopol.*
82		*Lupinus gussoneaus* J.Agardh.	*T scap*	*Steno–Medit.*
83		*Ononis spinosa* L. subsp. *antiquorum* (L.) Arcang.	*Ch suffr*	*Euri-Medit.*
84	Linaceae	*Linum usitatissimum* L. subsp. *angustifolium* (Huds.) Thell.	*H bienn/H scap (T scap)*	*Euri-Medit.-Subatl.*
85	Lythraceae	*Punica granatum* L.	*P scap*	*SW-Asiat.*
86	Malvaceae	*Malva sylvestris* L.	*H scap (T scap)*	*Subcosmop.*
87	Molluginaceae	*Corrigiola litoralis* L.	*T scap*	*Medit.–Atlant.*
88	Moraceae	*Ficus carica* L.	*P scap*	*Medit.–Turan.*
89	Myrtaceae	*Eucalyptus* sp. pl. Dehn.	*P scap*	*Australia (coltivate)*
90		*Myrtus communis* L.	*P caesp*	*Steno-Medit.*
91	Oleaceae	*Olea europaea* L.	*P caesp/P scap*	*Steno-Medit.*
92		*Phillyrea latifolia* L.	*P caesp (P scap)*	*Steno-Medit.*
93	Onagraceae	*Epilobium hirsutum* L.	*H scap*	*Subcosmop.*
94	Orchidaceae	*Ophrys apifera* Huds.	*G bulb*	*Medit.-Atlant. (Euri.)*
95	Papaveraceae	*Fumaria capreolata* L.	*T scap*	*Euri-Medit.*
96		*Papaver rhoeas* L.	*T scap*	*E-Medit.*
97	Pinaceae	*Pinus* sp. pl.	*---*	*---*
98	Plantaginaceae	*Linaria pelisseriana* (L.) Mill.	*T scap*	*Medit.-Atlant.*
99		*Plantago coronopus* L.	*T scap/H bienn/H ros*	*Euri-Medit.*
100		*Veronica anagallis-aquatica* L.	*H scap (T scap)*	*Cosmop.*
101	Poaceae	*Avena barbata* Pott ex Link	*T scap*	*Euri–Medit.–Turan.*
102		*Cynodon dactylon* (L.) Pers.	*G rhiz/H rept*	*Termo-Cosmop.*
103		*Lolium rigidium* Gaudin	*T scap.*	*Paleosubtrop.*
104	Polygonaceae	*Polygonum aviculare* L.	*T rept*	*Cosmop.*
105		*Rumex crispus* L.	*H scap*	*Subcosmop.*
106	Primulaceae	*Lysimachia arvensis* (L.)	*T rept*	*Subcosmop.*
107		*Lysimachia foemina* (Mill.) U.Manns & Anderb.	*T rept*	*Subcosmop.*
108	Pteridaceae	*Adiantum capillus-veneris* L.	*G rhiz*	*Pantropic. e -subtropic.*
109	Ranunculaceae	*Anemone hortensis* L.	*G rhiz*	*S-Medit.*
110		*Clematis flammula* L.	*P lian (H scap)*	*Euri–Medit.*
111		*Clematis vitalba* L.	*P lian*	*Europ.-Caucas.*
112		*Ficaria verna* Huds.	*G bulb/H scap*	*Eurasiat.*
113		*Ranunculus macrophyllus* Desf.	*H scap*	*SW-Medit.*
114	Resedaceae	*Reseda luteola* L.	*H scap/T scap*	*Circumbor*
115	Rhamnaceae	*Ziziphus jujuba* Mill.	*P caesp/P scap*	*SE-Asiat.*
116	Rosaceae	*Agrimonia eupatoria* L.	*H scap*	*Subcosmop.*
117		*Crataegus laevigata* (Poir.) DC.	*P caesp (P scap)*	*Centroeurop.*
118		*Crataegus monogyna* Jacq.	*P caesp (P scap)*	*Paleotemp.*
119		*Potentilla reptans* L.	*H ros*	*Subcosmop.*
120		*Poterium sanguisorba* L.	*H scap*	*Subcosmop.*
121		*Prunus spinosa* L.	*P caesp*	*Europ.-Caucas.*
122		*Rosa canina* L.	*NP*	*Paleotemp.*
123		*Rubus ulmifolius* Schott	*P caesp*	*Euri-Medit.*
124	Rubiaceae	*Galium aparine* L.	*T scap*	*Eurasiat.*
125	Rutaceae	*Ruta chalepensis* L.	*Ch suffr*	*S-Medit.*
126	Scrophulariaceae	*Scrophularia trifoliata* L.	*H scap*	*Endemism*
127		*Verbascum creticum* (L.) Kuntze	*H bienn*	*SW-Medit.*
128	Smilacaceae	*Smilax aspera* L.	*P lian (NP, G rhiz)*	*Paleosubtrop.*
129	Solanaceae	*Hyoscyamus niger* L.	*T scap/H bienn*	*Eurasiat.*
130		*Solanum nigrum* L.	*T scap*	*Cosmop. Sinantrop.*
131	Tamaricaceae	*Tamarix africana* Poir.	*P scap./caesp*	*Steno-Medit.-Occid.*
132	Thymelaeaceae	*Daphne gnidium* L.	*P caesp*	*Steno-Medit.–Macarones.*
133		*Thymelaea hirsuta* (L.) Endl.	*NP/Ch suffr*	*S-Medit.–W-Asiat.*
134	Ulmaceae	*Ulmus minor* Mill.	*P caesp./P scap.*	*Europ.–Caucas.*
135	Urticaceae	*Urtica dioica* L.	*H scap*	*Subcosmop.*
136		*Urtica pilulifera* L.	*T scap (H bienn)*	*S-Medit*
137	Violaceae	*Viola alba* Besser subsp. *dehnhardtii* (Ten.) W.Becker	*H ros*	*Euri-Medit.*

Life forms: *P*, phanerophytes; divided in *caesp*, caespitose; *lian*, lianose; NP, nano-phanerophytes, *scap*, scapose; *succ*, succulent. *Ch*, chamaephytes; divided in *frut*, frutescent and, *suffr*, suffrutescent. *G*, geophytes, divided in bulb, bulbous, rad, radicigemma, rhiz, rhizome, and tub, tuber. *H*, hemi-cryptophytes, divided in bienn, biennal, caesp, caespitose, rept, reptant; *ros*, rosulate, and *scap*, scapose. *T*, therophytes, divided in *scap*, scapose, and *rept*, reptant.

## Data Availability

Not applicable.
